# A case report of a teenager with severe hand, foot, and mouth disease with brainstem encephalitis caused by enterovirus 71

**DOI:** 10.1186/s12887-019-1428-4

**Published:** 2019-02-13

**Authors:** Ying-Fu Chen, Lan Hu, Feng Xu, Cheng-jun Liu, Jing Li

**Affiliations:** Intensive Care Unit, Key Medical Laboratory of Pediatrics, Chongqing Health Bureau, Ministry of Education; Key Laboratory of Child Development and Disorders, Children’s Hospital of Chongqing Medical University, Chongqing, People’s Republic of China

**Keywords:** HFMD, Enterovirus 71, Brainstem encephalitis, Teenager patient

## Abstract

**Background:**

Hand, foot, and mouth disease (HFMD) is an acute viral infection occurring mostly in infants and children. Enterovirus 71 (EV71) infection mostly occurs in children < 5 years of age. Severe cases, however, are usually encountered in children under the age of 3 years, and exceedingly rare in teenagers > 14 years and adults.

**Case presentation:**

We report a rare case of HFMD in a 16-year-old male teenager residing in Chonqing, China. The clinical presentation was typical of HFMD and included vesicular lesions and oral mucosal ulcers, macular and vesicular lesions on palms and soles. He developed severe neurological complications that were suggestive of brainstem encephalitis. EV71 RNA was detected in the patient’s faecal samples by reverse transcription-polymerase chain reaction. Specific IgM antibody to EV71 was detected in both serum and cerebrospinal fluid by ELISA. Gamma immunoglobulin therapy at 25 g/day was administered for 2 days, along with methylprednisolone, mannitol, ganglioside, and creatine phosphate sodium. The patient showed neurological improvement and recovered completely in 1 month.

**Conclusions:**

This case indicates that EV71 infection may cause HFMD in teenagers with potentially severe neurological involvement. Clinicians should be aware of the possibility of HFMD occurring in adults and teenagers as prompt treatment could be life-saving in these patients.

## Background

Hand, foot, and mouth disease (HFMD) is an acute viral infection occurring mostly in infants and children. Its name is derived from the typical presence of oval vesicular lesions on the hands and feet, and painful oral mucosal ulcerations. The major etiological agents of HFMD are Human Enterovirus A (HEVA), most commonly, Enterovirus 71 (EV71) and Coxsackievirus A16 (CVA16), although several other viruses such as EV-D68 and CVA6 have also been implicated [1]. EV71 infection mostly occurs in children < 5 years of age. Severe disease, however, is usually encountered in children under the age of 3 years. Severe cases are exceedingly rare in teenagers > 14 years and adults. EV71 has been associated with severe and sometimes fatal neurological complications such as aseptic meningitis, acute flaccid paralysis, encephalitis, and neurogenic pulmonary edema. There are very limited reports of neurological manifestations in an adult with EV71 infection. In this study, we report a 16-year-old teenage boy with HFMD due to EV71 infection with severe neurological complications.

## Case presentation

A 16-year-old male was admitted to the Department of Infectious Diseases at the Children’s Hospital of Chongqing Medical University, Chongqing, P. R. China, on June 30, 2014 with a history of fever, skin rash over hand and feet, headache, and weakness in lower limbs over the past 4 days. The patient also had intraoral and throat pain, and non-projectile vomiting 3 days prior to admission. Two days prior to admission, the patient developed drowsiness, startle, hand tremor, urinary incontinence, and progressive deterioration in consciousness. He reported recent contact with a HFMD. Medications were limited to recent use of over-the-counter analgesics. The patient’s body temperature was 36.8 °C, respiratory rate was 25/min, pulse rate 98 beats/min, and blood pressure was 124/76 mmHg. Vesicular lesions and ulcers were present in the oral mucosa, and macular and vesicular lesions were present on palms and soles.

The patient was drowsy and non-verbal, but was responding to painful stimuli. He showed left-sided facial paralysis. The left nasolabial fold was flat and there was drooping of the mouth to the left side. The pupils were equal in size (diameter: 4 mm) and the pupillary light reflex was bilaterally symmetrical. Neck resistance was normal. The left upper and lower limbs showed reduced muscle strength (grade III–IV). The muscle strength in right limb was normal. Abdominal reflex and cremasteric reflex were normal. Pathological reflexes (e.g., Babinski, Chaddock, Oppenheim, Gordon) were negative. The rest of the physical findings were unremarkable.

Results of blood test were as follows: White blood cell count, 10.82 × 10^9^; neutrophils, 92%; C-reactive protein, 80 mg/L, and blood glucose, 7 mmol/L. Findings of cerebrospinal fluid (CSF) examination were as follows: Total number of cells, 188 × 10^6^/L; nucleated cells, 44 × 10^6^/L; monocytes 37 × 10^6^/L; multinucleated cells 7 × 10^6^/L; protein, 0.65 g/L; glucose, 5.74 mmol/L, and chlorides, 120.4 mmol/L.

IgM levels were quantified using ELISA kit (Cat No. 20143400198, Wantai Biopharm Inc., China). The CSF and serum tested positive for IgM antibody to EV71, but negative for IgM antibodies against Enterovirus, Herpes simplex virus, Cossack virus, and measles virus. EV71 RNA, but not CVA16, was detected in the patient’s faeces by reverse-transcriptase-polymerase chain reaction (RT-PCR) (Cat No. 20133400621, SANSURE Biotech Inc., China). All tests were performed in the clinical laboratory at the Children’s Hospital of Chongqing Medical University, Chongqing, P. R. China. Eight hours after admission, the patient showed progressive loss of consciousness and was transferred to the paediatric intensive care unit (PICU). He was in a coma and exhibited shallow breathing (30–40 breaths per minute). Pupils were sluggishly responsive to light with mild anisocoria (OD = 3 mm and OS = 4 mm). The patient showed no response to painful stimuli, and thus the muscle strength was not detected. The status of abdominal, cremasteric, and pathological reflexes was identical to that at the time of hospital admission. Based on the above clinical symptoms, a diagnosis of severe HFMD with brain stem encephalitis was established by specialists in the Department of Neurology and the Department of Infectious diseases.

The patient was administered mannitol (5 mL/kg/dose, q4h) to reduce the intracranial pressure, ganglioside for neurological recovery, creatine phosphate sodium for providing heart muscle energy, methyl prednisolone for reducing inflammation and cerebral edema, and potassium sodium dehydroandroan drographolide succinate as antiviral therapy. The patient was also administered midazolam (5 mg) twice daily on days 1 and 2 after admission to prevent agitation. From day 2, the patient received gamma immunoglobulin therapy (25 g/day) for 2 days.

Head CT performed on day 3 following admission showed signs of pineal calcification; no other obvious abnormality was observed in other parts. The patient was unconscious and comatose during EEG examination performed on day 3. The results were indicative of diffuse encephalopathy with mixed delta and theta wave activity in the range of 1–4 Hz, which indicated abnormal brain function.

The patient showed improvement and was transferred back to the Department of Infectious Diseases on day 4 following admission, and the same treatment was continued, except for gamma immunoglobulin therapy. On day 5, the patient regained consciousness, but showed paroxysmal increase in muscle tension in the limbs (mainly on the right side).

Head MRI performed on day 9 was normal. The patient showed progressive improvement and was able to walk with an unsteady gait; he was discharged from the hospital on day 10 following admission.

Two weeks after discharge, the patient still walked with an unsteady gait, but showed full recovery 1 month after discharge (normal limb muscle strength and tone, movement, and intelligence).

## Discussion and conclusions

HFMD is an acute infectious disease caused by a group of enteroviruses, among which EV71 and CVA are the most common pathogens [2]. The symptoms are generally mild and self-limiting. The main clinical manifestations are fever, rash, and ulcers in oral mucosa, over hands, feet, and buttocks. A small proportion of paediatric patients are known to develop severe complications such as meningitis, encephalitis, encephalomyelitis, neurogenic pulmonary edema, and circulatory failure [3]. Since the first report in the year 1958, HFMD outbreaks have been reported in East and Southeast Asia [4–9]. Since 2008, several epidemics of HFMD have been reported in China [10]. HFMD caused by EV71 may be associated with severe, potentially life-threatening complications in children, such as brainstem encephalitis, aseptic meningitis, encephalitis, flaccid paralysis, heart failure, and lung failure [11]. Approximately 10–30% of the hospitalized patients during EV71-associated HFMD epidemics in Asia reportedly developed a spectrum of neurological complications [3, 8]. Brainstem encephalitis, a distinctive form of encephalitis with typical neuropathological characteristics, has been the hallmark of severe HFMD during EV71 epidemics in Asia, which began in the late 1990s. Approximately 45% of mild, 80% of severe, and 93% of critically-ill paediatric cases of HFMD in China are known to be caused by EV71 infection [12]. Moreover, EV71 infection also caused three deaths among HFMD children in Singapore in the year 2001 [13].

The patient in the present study had typical clinical features of severe HFMD. The patient showed hemiplegia, bilateral pupil asymmetry, shallow breathing, and sluggish pupillary light reflex. The diagnosis of brain stem encephalitis was established based on the above clinical symptoms by specialists in the Department of Neurology and the Department of Infectious diseases, although the CT and MRI showed no abnormality at that moment. EV71 RNA was detected in faeces by RT-PCR. The Chinese National Centre for Disease Control and the guidelines for the prevention and control of Hand, Foot and Mouth Disease in China recommend the use of RT-PCR for detection of virus in the stool and oropharyngeal secretions due to the high positive rate. Moreover, detection of EV71 in stool samples by RT-PCR is widely used in clinical settings to monitor the development of HFMD [14]. In addition, ELISA for anti-EV71 IgM levels in CSF and blood samples are also used to confirm the viral infection [14, 15].

HFMD occurs mostly in children under the age of 5 years [4, 16]. However, adult cases of HFMD caused by CVA16 virus have been reported at the age of 21, 35, and 37 years [17].

Severe and critically-ill patients with HFMD caused by EV71 are mostly under the age of 3 years, and are rarely seen in children above the age of 14 years [12]. The patient in this report was 16 years and 8 months old, with a definite history of exposure to HFMD, and exhibited severe symptoms of HFMD. The median duration from onset to diagnosis of HFMD in China has been reported to be 1.5–3.5 days, while median duration from onset to death has been reported to be 3.5 days or 0.5 days after diagnosis of HFMD [12, 16]. In the present report, the patient was diagnosed with HFMD on the fourth day of onset, and developed neurological manifestations on the third day of onset, which is consistent with the progression of HFMD in children in the age-group of 1–3 years.

World Health Organization as well as HFMD epidemic countries have developed guidelines for the diagnosis and treatment of HFMD, including the staging and classification of HFMD. Different treatments are employed for respective phases of HFMD [18, 19]. According to the above guidelines, in this case, gamma immunoglobulin therapy that was initiated immediately after the onset of severe neurological complications, combined with other treatments, resulted in the rapid improvement of the manifestations. Milrinone is a type III phosphodiesterase inhibitor with both inotropic and vasodilator effects [20]. It was shown to reduce mortality in patients with severe HFMD with cardiopulmonary collapse in a small randomised controlled open-label trial [21]. We also found that in the treatment of severe HFMD, milrinone in combination with hydrochloride esmolol helps reduce the heart rate and maintain cardiopulmonary function.

This case illustrates that EV71 infection may cause HFMD accompanied by severe neurological manifestations in teenagers. The course of the disease and the associated complications in teenagers are similar to those in infants and children. Clinicians should be aware of the possibility of HFMD occurring in adults and teenagers, as prompt treatment could be invaluable in reducing complications and saving lives.

## Abbreviations

CSF: Cerebrospinal fluid
CVA16: Coxsackievirus A16
EV71: Enterovirus 71
HEVA: Human Enterovirus A
HFMD: Hand, foot, and mouth disease
PICU: Paediatric intensive care unit
RT-PCR: Reverse-transcriptase-polymerase chain reaction
